# AI-based virtual patient interactions improve professional language proficiency in international nurses

**DOI:** 10.3205/zma001868

**Published:** 2026-09-15

**Authors:** Sebastian Ullrich, Jan-Marcus Haus, Sandra Lamb, Nicole Hermandung, Tom Friemel, Olav Stolze, Sabine Schmidt, Olha Demianiuk, René Werner, Parisa Moll-Khosrawi, Rüdiger Schmitz

**Affiliations:** 1 Städtisches Krankenhaus Kiel, Department of Internal Medicine III, Kiel, Germany; 2 University Medical Center Hamburg-Eppendorf, Department of Anaesthesia, Hamburg, Germany; 3 Städtisches Krankenhaus Kiel, Directorate of Nursing, Kiel, Germany; 4 DRK Akademie SH, Kiel, Germany; 5 Städtisches Krankenhaus Kiel, Department of Internal Medicine I, Kiel, Germany; 6 casuu GmbH, Hamburg, Germany; 7 University Medical Center Hamburg-Eppendorf, Institute for Applied Medical Informatics, Hamburg, Germany; 8 University Medical Center Hamburg-Eppendorf, Institute for Computational Neuroscience, Hamburg, Germany; 9 University Medical Center Hamburg-Eppendorf, Center for Biomedical Artificial Intelligence (bAIome), Hamburg, Germany

**Keywords:** international healthcare personnel, professional language training, nursing education, virtual interaction training, virtual patients

## Abstract

**Objectives:**

International healthcare personnel face significant challenges in language acquisition. We investigated whether virtual patient interaction (VI) training can support professional integration and clinical language proficiency.

**Methods:**

In an explorative controlled study, 25 international nursing staff undergoing professional qualification measures in Germany were included. The intervention group (n=13) received access to casuu, a digital simulation tool providing interactive communication scenarios with virtual patients and colleagues. The control group (n=12) followed standard language training without VI. Both groups received 600 hours of training for the B2 for Nursing exam, as required for professional licensure in several German states, at the same language school using the same textbook. The primary outcome was the fail rate at the first attempt, with effects on different skill dimensions assessed in addition. The clinical supervisors were interviewed 12-14 months after the arrival.

**Results:**

The fail rate in the B2 for Nursing exam was reduced from 66.7% in the control group to 25.0% in the intervention group (relative reduction 62.5%). Competence increased primarily in the skill dimension “active speaking”. Attrition rates did not increase (1/13 vs. 2/12). Clinical superiors reported increased confidence and competence in team and patient interactions in the intervention group.

**Conclusion:**

Virtual interaction-based training improves the acquisition of professional language and clinical communication skills of international nurses. It integrates with conventional teaching and does not increase dropout rates.

## Zusammenfassung

**Ziele:**

Internationales Gesundheitspersonal steht beim Spracherwerb vor erheblichen Herausforderungen. Wir untersuchten, ob ein Training mit virtuellen Patient:inneninteraktionen (VI) die berufliche Integration und den Erwerb der klinischen Sprachkompetenz unterstützen kann.

**Methoden:**

Kontrollierte Studie mit 25 international ausgebildeten Pflegefachpersonen im Anerkennungsverfahren. Beide Gruppen absolvierten einen 600-stündigen Vorbereitungskurs für die Prüfung B2 Pflege an derselben Sprachschule und mit demselben Lehrbuch. Die Interventionsgruppe (n=13) erhielt zusätzlich Zugang zu casuu, einem digitalen Simulationstool, das interaktive Kommunikationsszenarien mit virtuellen Patient:innen und Kolleg:innen bereitstellt. Die Kontrollgruppe (n=12) absolvierte den Sprachkurs ohne ergänzendes VI. Primärer Endpunkt war die Durchfallquote beim ersten Prüfungsversuch; zusätzlich wurde die Wirkung des Trainings auf verschiedene Kompetenzdimensionen untersucht. Außerdem wurden die Praxisanleiter:innen der Teilnehmer:innen 12-14 Monate nach der Ankunft interviewt.

**Ergebnisse:**

Die Durchfallquote in der Prüfung B2 Pflege sank von 66,7% in der Kontrollgruppe auf 25,0% in der Interventionsgruppe, was einer relativen Reduktion um 62,5% entspricht. Die Kompetenz nahm vor allem in der Dimension „aktives Sprechen“ zu. Die Abbruchquoten stiegen nicht an (1/13 vs. 2/12). Klinische Vorgesetzte berichteten von erhöhter Sicherheit und Kompetenz in Team- und Patient:inneninteraktionen in der Interventionsgruppe.

**Schlussfolgerung:**

Ein auf virtuellen Interaktionen basierendes Training verbessert den Erwerb berufsbezogener Sprachkompetenz und klinischer Kommunikationsfähigkeiten bei internationalen Pflegefachpersonen. Es lässt sich in den konventionellen Unterricht im Rahmen des Anerkennungsverfahrens integrieren und erhöht die Abbruchquoten nicht.

## Introduction

The shortage of skilled nursing personnel in Germany and many countries is acute and persistent. A central countermeasure is the integration of international nursing staff, with a recent net immigration of 36,000 persons in Germany (+36% to previous year [1]). Since 2021, the increase in employment within nursing professions has been entirely driven by immigration, while the number of nationals in these professions is actually declining [2].

Language barriers and lack of specific language training are repeatedly reported as the most important barriers in this process [3], [4], [5]. Limited language proficiency correlates with work-life balance problems [6] and stress [7] as early warning signals for job discontinuation [8]. As a result, 32.1% of employment contracts with internationally recruited nurses end within the first 12 months [9], implying limited language proficiency as a potentially relevant factor in job discontinuation. Among those who continue, high failure rates in Kenntnisprüfung (KP) and/or professional language examinations can delay the process by months until full professional recognition and clinical readiness are reached. The authors are aware of anecdotal evidence of first attempt failure rates of up to 90% for certain examinations and cohorts.

 There is reason to assume that language barriers may adversely affect the quality of medical care – an effect that is, at minimum, well-documented from the patient's perspective [10]. The available data for the effect of language barriers on the side of the healthcare provider is limited. However, there is plenty of data that, reversing the situation and considering patients who are native in a foreign language, suggests that the effect of limited language proficiency on patient safety is substantial [11], [12], [13].

Efforts to facilitate a qualitative, quick and reliable (professional) language acquisition are thus of the utmost importance in times of labour shortage and international migration. This study evaluates the effect of a novel, AI-based virtual training scheme on the professional language proficiency of international nurses.

## Material and methods

A total of 25 internationally trained nursing staff undergoing professional qualification measures were enrolled (intervention group, n=13; control group, n=12). Both groups attended parallel 6 month, 600-hour qualification courses at the same language school (DRK Akademie Schleswig-Holstein, Kiel) and using the same textbook, ensuring consistency in teaching quality and curriculum. 92% of the participants (23/25) originated from the same home country, Tunisia, the others from Nepal (n=1) and Ukraine (n=1). All entered training with comparable initial German language proficiency (CEFR level B1) and comparable demographics (cf. table 1 (Tab. 1)).

The intervention group received access to casuu (casuu GmbH, Hamburg, Germany, [https://casuu.com]), a digital simulation platform that provides interactive, AI-supported communication scenarios simulating real-life clinical situations with virtual patients and colleagues [14]. Usage was mostly self-directed but encouraged, with the exception of three approximately two-week phases of structured integration into the classroom-setting including assigned homework and integrated debriefing sessions. The 15-20 minute debriefing sessions involved individual students presenting the transcripts of their VIs to the class via a projector. Guided by the teacher, the class then discussed newly emerging lexical fields, vocabulary gaps, and whether the expressions used had already been covered in class. The control group attended standard language training that did not include VI training, but otherwise did not differ in terms of curriculum or contents.

In parallel to the language courses, participants in both groups underwent clinical onboarding on the wards of the Städtisches Krankenhaus Kiel (SKK). Over the course of the program, the ratio of clinical work to language training gradually shifted from approximately 1:4 days per week at the beginning to 4:1 toward the end. The structure and intensity of language teaching and practical clinical work were identical for both groups.

The primary outcome was the first-attempt failure rate at the B2 for Nursing professional language examination (telc^®^ Deutsch B2 Pflege) as required for professional licensure in several German states. Both groups conducted this exam at the same, external, institution (Inlingua Sprachcenter Kiel, Kiel) approximately 8 months after arrival. The overall score at the exam was recorded as a secondary outcome. Furthermore, training effects across different language skill domains – receptive skills (reading and listening), writing and speaking – were analyzed using the anonymized section-level results from the same examination. Attrition rates from the qualification program were also recorded. The primary outcome was then analyzed both for all enrolled participants (intention-to-treat, ITT) and for those who continued until the language examination (per-protocol, PP).

To explore clinical instructors’ perspective on the onboarding and integration of the nursing staff, semi-structured interviews were conducted with four clinical supervisors (Praxisanleiter*innen) at SKK. Each interview lasted between 30 and 45 minutes and was conducted by a single interviewer, who documented the interviews in structured summary notes. Prior to the interview, participants provided informed consent to anonymous processing of their responses. The summary of the interviews was then reviewed and verified by three independent readers to ensure accuracy and completeness. The thematic structure of the interview guide provided a deductive framework, within which the participants’ responses were condensed into categories. The interview guide is available in the attachment 1 . 

## Results

Of those who attended the B2 for Nursing exam, 25.0% (3 out of 12) failed in the intervention group as compared to 66.7% (6 out of 9) in the control group, corresponding to a relative reduction of 62.5% (p=0.028) per protocol.

During the course of the training, 1 participant from the intervention group terminated their contract with SKK and dropped out of the training. In the control group, this was the case for two nurses. In addition, in the control group, 1 person took their B2 exam while on vacation at home, effectively violating the study protocol. To avoid overestimating the effect of the intervention, this case was conservatively counted as a pass. Therefore, in the ITT analysis, only 30.8% (4 out of 13) in the intervention group were counted as “failed” (failed at the exam or terminated early), as compared to 66.7% (8 out of 12) of those who received the traditional training alone. This corresponds to a relative reduction in the fail rate of 53.8% (p=0.036).

Overall exam scores were 140.25±17.15 (125.50±14.89) in the intervention (control) group (p=0.030). With only minor and insignificant differences in “receptive” and “writing” skill dimensions, the difference was mainly attributed to “active speaking” (arithmetic mean 88.58 ± standard deviation 8.83 versus 77.39±9.84, p=0.009).

The attrition rate within the qualification programs was 1/13 in the VI group vs. 2/12 in the control group, indicating that the additional training did not increase dropout risks. The intensity of the VI training was higher in phases when it was systematically integrated into the classroom-based course by the structured debriefings (62-100% weekly active users) as compared to other weeks (0-62%).

Qualitative feedback from individual supervisors suggested that VI-trained participants exhibited greater confidence in clinical communication and interpersonal interactions. In a round of semi-structured interviews of clinical supervisors, all four interviewees noted a marked reduction in language anxiety and an increase in communicative confidence. Nurses were consistently described as speaking more fluently, engaging more actively in team interactions, and performing more capably in patient-facing situations. All respondents drew a direct link between language competence and patient safety. Higher language proficiency in the intervention group, in the view of the clinical supervisors, also helped in facilitating a faster onboarding process as witnessed by the clinical supervisors. 

## Conclusion and outlook

The results show that virtual interaction (VI) training can substantially improve professional language acquisition in international healthcare personnel. Integration of VI training did not increase dropout rates and was particularly effective on active speaking skills.

This study has a number of limitations: First, the overall sample size is limited. Second, a larger effect may be hypothesized if starting earlier and in the home country. Both will be the focus of a larger, government-funded study (V-PREP-CARE). Third, participants were not restricted from completing the exercises collaboratively, assumingly blurring data on user activity in phases without classroom-integrated usage. Fourth, although not actively informed, it can be assumed that the clinical supervisors (Praxisanleiter) were aware of which participants underwent additional VI training. The results from the semi-structured interviews are therefore potentially subject to observer bias. In contrast, the examiners of the B2 for Nursing examination were blinded to participants’ group allocation. Fifth, given the summarized character of the qualitative data, findings of the interviews reflect interpreted content rather than literal transcribed accounts of the interviewees. They should therefore be understood more as exploratory rather than exhaustive. Sixth, anonymized test results reported by the examination body distinguished only the skill dimensions “receptive”, “writing”, and “active speaking”, effectively merging listening and reading into a single category. Nevertheless, the data suggest that listening and reading comprehension may be particularly relevant targets for future refinements of the training program.

Virtual interaction training holds potential for reducing systemic costs and time until clinical readiness, enhancing the efficiency of workforce integration, and improving healthcare delivery in the context of labor shortage and workforce migration. It should be considered as a potentially important element of sustainable and future-ready strategies for addressing the nursing workforce shortage in Germany and beyond.

## Notes

### Author contributions

US and JMH share first authorship.

PMK and RS share last authorship.

### Authors’ ORCIDs

- Jan-Marcus Haus: [0000-0003-0012-4137]
- Tom Friemel: [0009-0001-1250-9256]
- Olha Demianiuk: [0009-0000-6784-5305]
- René Werner: [0000-0002-2296-3305]
- Parisa Moll-Khosrawi: [0000-0003-2024-0020]
- Rüdiger Schmitz: [0000-0002-2414-9817]

## Acknowledgements

We are grateful towards the nurses who took part in this study, their teachers and staff at DRK Akademie SH, as well as their supervisors and staff at Städtisches Krankenhaus Kiel. We acknowledge support by Inlingua Kiel and telc^®^ gGmbH. We thank Sebastian Zimmermann of telc gGmbH for the compilation and anonymization of the exam results. 

## Competing interests

US, OS and SL have roles in the follow-up study V-PREP-CARE. RW is an advisor to casuu GmbH [https://casuu.com/] on a pro bono basis and without financial interests. RS is a co-founder and shareholder of casuu. OD is a student assistant to the company. 

The other authors declare that they have no competing interests. 

## Supplementary Material

Interview guide: Clinical instructors and international nurses

## Figures and Tables

**Table 1:**
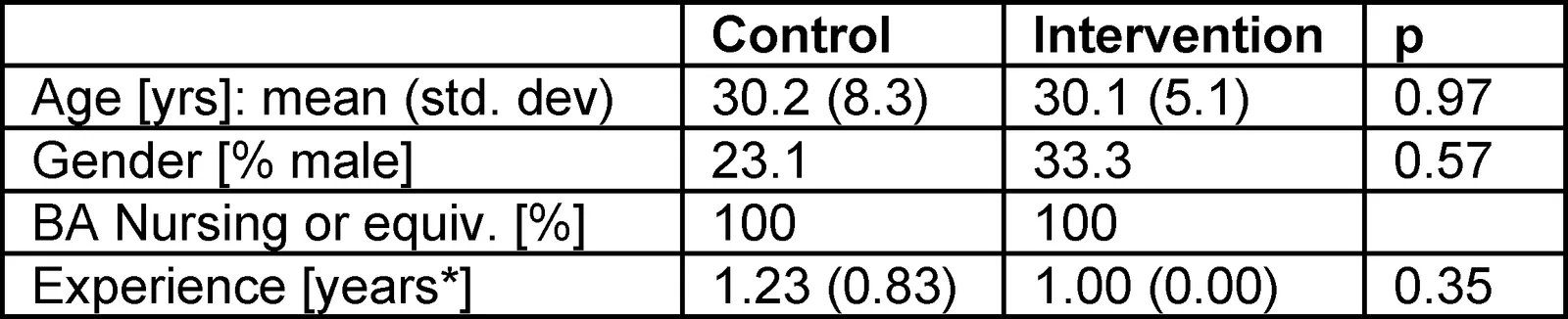
Demographics, qualification level and professional experience Note that for the professional experience, years in nursing in a hospital setting were considered, and data rounded to full years.
