# Supplementary material for: AI-based virtual patient interactions improve professional language proficiency in international nurses
Source: GMS J Med Educ. 2026 Sep 15;43(6):Doc74. doi: 10.3205/zma001868 (PMC13617267; doi:10.3205/zma001868)
Supplement: Supplementary file 1 [file JME-43-74-s-001.pdf]

Attachment 1: Interview guide: Clinical instructors and international nurses

A. Opening and background

1. Welcome, introductions, context
   "Thank you very much for taking the time for this interview. Today we'll be discussing your experiences as a clinical instructor working with international nursing professionals, particularly with a group that has undergone more intensive job-specific language training."
2. Purpose and procedure
   "The purpose of this conversation is to better understand how you experience the onboarding, guidance, and integration of these international nursing professionals and what role the language training plays in this process. The interview will take about 30–45 minutes."
3. Data protection and consent
   Briefly explain: voluntary participation, anonymization, confidentiality, and the right to withdraw consent.
4. Icebreaker
   "To start off: Could you briefly describe your role in your department and how long you've been working as a clinical instructor?"

B. Background: General experiences with international nursing staff

1. Initial level of experience

"How long have you been working with internationally recruited nurses, and to what extent do you interact with these colleagues in your daily work?"

"How would you describe your overall experience with international nurses so far?"

2. Role as a clinical instructor

"What tasks do you typically take on when training and supporting international nurses?"

"In your view, how does the training of international nurses differ from that of new colleagues who were trained in Germany?"

3. Typical situations/ examples

"Can you describe one or two situations that you find particularly typical of working with international nurses?"
"Were there any events that you remember as being particularly positive or particularly challenging?"

Attachment 1 to Ullrich S, Haus JM, Lamb S, Hermandung N, Friemel T, Stolze O, Schmidt S, Demianiuk O, Werner R, Moll-Khosrawi P, Schmitz R. *AI-based virtual patient interactions improve professional language proficiency in international nurses.* GMS J Med Educ. 2026;43(6):Doc74. DOI: 10.3205/zma001868

C. Specific cohort with intensive language training

(If necessary, briefly define at the beginning of this section what is meant by “intensive language training”: duration, format, Casuu, job-specific content.)

1. Distinction from other groups

“You are currently working with a group of international nursing professionals who have undergone a more intensive job-specific language training. In your view, how does this group differ from other international colleagues you have worked with previously?”

Follow-up question: “How exactly do you identify these differences?”

2. Initial overall impression

“How would you describe your first impression of this group that underwent intensive language training when the colleagues first arrived on the ward?”

“Did this impression change during the orientation period? If so, how?”

D. Perceived language and communication skills

1. Communication within the team

“How do you experience the communication of these nurses within the team, for example during handoffs, brief discussions, or when interacting with doctors and therapists?”

“Are there differences compared to international nurses without this intensive language training?”

2. Communication with patients and family members

“How would you assess their ability to communicate effectively in direct contact with patients and family members (providing information, giving instructions, offering advice, and conducting conversations in stressful situations)?”

3. Documentation and Written Communication

“Are there situations where you notice that colleagues particularly benefit from the language training? Can you give examples?”

“How do you perceive your colleagues when it comes to written documentation, e.g., care planning, progress notes, reports?”

“Are there differences here compared to other international nurses who have had no language training or less intensive training?”

4. Typical strengths and remaining difficulties

"What language strengths do you observe particularly frequently in this group?"
"Where do you still see significant challenges despite intensive language training?"

E. Impact on guidance, orientation, and integration

1. Onboarding process

"How does this group's language proficiency affect the onboarding process?"

(For example, on the duration of onboarding or the need for support from you as a clinical instructor?)

"Do you feel that you can structure your guidance differently than you would for international nurses without intensive language training? If so, how exactly?"

2. Role of the clinical instructor and workload

"What impact does the intensive language training have on your own role as a clinical instructor? Do you feel more relieved, more challenged, or does it have little impact?"

"In which situations do you notice most strongly that your colleagues' communication skills make your guidance easier or more difficult?"

3. Team dynamics and collaboration

"How is this group of international nurses perceived within the team?"

"Does the higher language proficiency (if you perceive it that way) have an impact on team atmosphere, collaboration, conflicts, or misunderstandings?"

4. Patient Safety and Quality of Care

"What impact do you see on patient safety and quality of care when international nurses have completed such intensive language training?"

"Can you think of specific situations in which good or poor language proficiency was particularly relevant?"

F. Evaluation of language training (Casuu)

1. Alignment with requirements

"To what extent do you consider the language training used to be appropriate for the requirements on your ward or in your department?"

"Are there areas of everyday nursing practice that, in your view, should be given greater consideration in the training?"

2. Perceived added value

"What positive effects do you attribute to the language training – in terms of the international nursing staff themselves, your work as a clinical instructor, and the team?"

"If you had to evaluate the program in a few sentences: What would you highlight?"

3. Suggestions for improvement

“What would you change or add to the current language support concept to ensure that international nursing staff are even better prepared for daily ward life?”

“What role could clinical instructors themselves play within such programs (e.g., feedback, practical tasks, feedback loops)?”

G. Structural conditions and support needs

1. Hospital and ward structures

“In your opinion, what structural or organizational factors in your facility facilitate the successful onboarding and integration of international nursing staff?”

“Which factors tend to make this more difficult?”

2. Support for clinical instructors

“What kind of support would you like to see for your role in guiding and integrating international nursing staff (e.g., time resources, training, materials, exchange formats)?”

“Are there any resources you are already familiar with or use that you have found helpful?”

H. Closing questions

1. Key message

“When you think about your experiences so far: What do you consider to be the most important insight regarding working with international nurses who have undergone intensive language training?”

2. Open-ended comments

“Is there anything else related to this topic that is important to you and that we haven't addressed yet?”

3. Thanks

“Thank you very much for your time and your openness. Your perspective is very valuable for understanding how language support and on-the-job training work together.”

Category overview for the survey of clinical instructors (Praxisanleiter, PA)

Kx	Main category	Description
K1	Language proficiency & communication skills	Covers all statements regarding oral and written language proficiency, proficiency in nursing terminology, any communication barriers, and the self-confidence of international nurses in using the language.
K2	Onboarding process & quality of training	Statements regarding changes to the onboarding process brought about by “Casuu,” the perceived quality and comprehensiveness (“depth”) of the practical guidance, and the role of language proficiency in the success of the guidance.
K3	Patient safety & quality of care	All statements regarding the perceived effects of language proficiency on the safety of patient care and the quality of nursing practices.
K4	Team integration & work environment	Statements on how international nursing staff are perceived within the team, and on the impact of language on team atmosphere, conflicts, and social integration.
K5	Review of Casuu	Direct evaluations of the language training program in terms of its added value regarding onboarding, suitability, and areas for improvement.
K6	Structural conditions & support needs	Comments on supportive and inhibiting structures in the hospital, as well as on the desired support for clinical instructors.

Attachment 1 to Ullrich S, Haus JM, Lamb S, Hermandung N, Friemel T, Stolze O, Schmidt S, Demianiuk O, Werner R, Moll-Khosrawi P, Schmitz R. *AI-based virtual patient interactions improve professional language proficiency in international nurses.* GMS J Med Educ. 2026;43(6):Doc74. DOI: 10.3205/zma001868

Detailed analysis by category

1: Language proficiency & communication skills

K1a - Improved language skills & reduced inhibitions

PA 1	Less anxiety about speaking, more fluent speech; nursing staff with Casuu experience are surprisingly proficient in the language
PA 2	Improves language proficiency by “at least one level”; reduces language inhibitions; Casuu builds confidence
PA 3	They feel much more confident speaking up; more active and open communication
PA 4	Clearly beyond the textbook level; strong (subjective) impression; a feeling that cannot be measured

All four clinical instructors unanimously observed a significant reduction in fear of speaking and inhibitions, as well as a higher level of language proficiency, among the group that had access to Casuu.

K1b - Remaining challenges

PA 1	Not specified
PA 2	German grammar remains a challenge; copy-and-paste errors in documentation
PA 3	Sometimes a lack of self-confidence despite having the skills; modesty as an obstacle
PA 4	Everyday conversations and colloquial language are challenging; natural speaking speed

Despite the improved professional language skills, everyday language, grammar, and speaking speed remain challenging in real-life situations.

K1c - Written documentation

PA 1	Some use AI support; in those cases, the results are very good
PA 2	Use cell phones as a tool; better language skills = better written documentation
PA 3	They write better and are more confident; their grammar used to be worse
PA 4	Somewhat difficult without help; very good with help

Writing skills have improved, but the nurses still rely on (digital) support.

K2: Onboarding process & quality of training

K2a - Accelerated and in-depth onboarding

PA 1	The higher the language proficiency, the earlier the nurses can engage with specialized content; better exam preparation
PA 2	Ideally, only slightly longer than for German-trained nurses; more specific guidance is possible
PA 3	Much easier, as the processes are better understood
PA 4	Improved language proficiency

All clinical instructors report that the onboarding of the international nurses has become significantly easier and more in-depth. A higher level of language proficiency allows for earlier access to specialized content.

K2b - Changes in the role of clinical instructors

PA 1	The clinical instructor can speak more naturally; has to worry less about language (feels less pressure)
PA 2	A significant challenge; more specific and detailed guidance is possible
PA 3	Little influence; instructions remain individualized
PA 4	Not a traditional clinical instructor role; a coordinating role

Opinions on the impact on the clinical instructor (Praxisanleiter, PA) role vary: from a reduction in workload (PA1) over improved possibilities (PA2) to a neutral impact (PA3). PA4 plays a special role (coordination).

K3: Patient safety & quality of care

K3a - Positive correlation between language and safety

PA 1	Better language comprehension → greater patient safety and higher quality of care
PA 2	Overall, patient safety is high
PA 3	Differences in language proficiency levels are evident in medication administration, fall prevention, and hygiene
PA 4	Significantly increased; improved information processing

All four clinical instructors see a clear positive correlation between higher language proficiency and patient safety.

K3b - Specific risk situations caused by language barriers

PA 1	Emergency situations
PA 2	Proper reporting of patient complaints
PA 3	Medication administration: insulin mix-up (long-acting vs. short-acting), similar-sounding medication or patient names
PA 4	Exam situations; Information processing under stress

Specific risk areas: emergency situations, medication safety, and communication under stress.

K4: Team integration & work environment

K4a - Language proficiency as a factor in integration

PA1	Better conversations with colleagues and break-time chats
PA2	Greater acceptance within the team: “Language brings people together”
PA3	A positive influence, especially in cases of conflict and misunderstandings
PA4	The better the language proficiency, the fewer conflicts

All clinical instructors describe language proficiency as a key factor in integration. A higher level of language proficiency reduces misunderstandings and strengthens team cohesion.

K5: Review of Casuu

K5a - Perceived added value

PA 1	“A very good project”; content determined in collaboration with the hospital; participants who have undergone training come across as confident
PA 2	“The difference is noticeable!”; shorter and more effective recognition
PA 3	“Great language training, very practical”; a more open atmosphere
PA 4	“An extremely good idea”; customizable; as good as it is used

All four clinical instructors gave “Casuu” a clearly positive rating. They particularly highlighted its practical relevance, customizability, and the measurable improvement in language proficiency.

K5b - Areas for improvement & suggestions

PA 1	Even more intensive language training; separate access for clinical instructors, if necessary
PA 2	More sessions offered after shifts
PA 3	More content on patient admission interviews, patient education, and active listening; an overview of the curriculum
PA 4	Give greater consideration to critical or emergency situations

A desire for greater depth, more situation-specific content (emergency situations, patient education), and greater involvement of clinical instructors in the program.

K6: Structural conditions & support needs

K6a - Contributing factors

PA1	Leave of absence for language instruction; Office for int. nurses
PA2	Many clinical instructors, our own international clinical instructor; collaboration with recognition (Anerkennung) school
PA3	Clinical instructor days, orientation week, cultural networking events
PA4	Team openness as a decisive factor

Protected time, dedicated points of contact, and an open team culture are considered key factors for success.

K6b - Barriers & wishes

PA 1	Not enough clinical instructors/mentors; more clinical instructor days are desired
PA 2	Staff shortage; More time off requested
PA 3	"Throw someone in at the deep end"; Request for an overview of the curriculum
PA 4	Team openness and awareness as areas for improvement; exchange formats that need further development

Staff shortages, a lack of infrastructure, and insufficient team awareness are cited as the main obstacles.
